# Severe Post-Herpetic Lumbar Plexopathy Responds to Pulse Intravenous Methylprednisolone: A Case Report With a Side Note on its Parallel Semiology to Diabetic Radiculoplexopathy and the Vascular Invasiveness of the Varicella-Zoster Virus

**DOI:** 10.7759/cureus.12171

**Published:** 2020-12-19

**Authors:** Hassan Kesserwani

**Affiliations:** 1 Neurology, Flowers Medical Group, Dothan, USA

**Keywords:** plexopathy, varicella-zoster

## Abstract

Post-herpetic lumbar plexopathy (post-herpetic segmental paresis), immune-mediated lumbar plexopathy and diabetic radiculoplexopathy (diabetic amyotrophy) have similar and seemingly parallel semiologies. The latter two conditions have an underlying microvasculitic pathological substrate that has shown potential (yet unproven) amelioration with immunodulatory therapy. These observations gave us the motivation to treat our patient with intravenous methylprednisolone for a profound proximal left leg weakness due to post-herpetic segmental paresis. In this case report, we outline in detail the clinical and electophysiolgical phenotype of this disease and we demonstrate the spectacular improvement of left leg power that had previously remained static for three months. In the discussion section, we review the vascular invasiveness of the varicella-zoster virus.

## Introduction

We have previously described a case of a post-herpetic brachial plexopathy in the Cureus Medical Journal. In that article we outlined the unique profile of post-herpetic segmental paresis due to the varicella zoster virus (VZV), including its prevalence, pathogenesis and signature neuroradiological profile of peripheral nerve hypertrophy and lack of contrast enhancement with gadolinium on magnetic resonance imaging (MRI) studies [1,2].

There are isolated case reports of therapeutic options and strategies [3]. The challenges of conducting clinical trials include the rarity of this condition, its relatively good prognosis when mild and evidence that pre-emptive treatment with anti-virals improves outcome [4,5].

Post-herpetic segmental paresis (post-HSP) has many parallels to diabetic radiculoplexopathy (formerly known as diabetic amyotrophy). Both are acute, painful, asymmetric and both manifest similar natural histories, with gradual improvement over the course of one to two years. An immune-mediated microvasculitis has been shown to explain these findings for diabetic radiculo-plexopathy [6,7]. Not surprisingly, non-diabetic radiculo-plexopathies have also been associated with a microvasculitic etiology [8].

Diabetic radiculoplexopathies have been treated with immunodulatory therapy such as intravenous immunoglobulins, intravenous steroids and plasma exchange [9,10,11,12,13].

We evaluated a patient with a profound post-herpetic lumbar plexopathy who presented to us three months after a herpetic shingles rash over the left lumbar L2 and L3 dermatomes. The patient's severe proximal left leg muscle weakness had reached a plateau. We seized upon this opportunity to proactively treat our patient with high doses of pulsatile intravenous steroids with the goal of expediting recovery. To our surprise, the patient demonstrated a striking and rapid improvement. In this case report, we document and localize our plexopathy clinically and electrophysiologically, and in the process, we are able to accurately and objectively outline the pattern of recovery. We also highlight the fact that despite the absence of gadolinium enhancement of the lumbar plexus on MRI, local microscopic inflammatory processes may still be active in these patients. This reasoning holds, as it is well established that there are ongoing humoral and cellular responses to varicella virus infections [14,15].

## Case presentation

We describe the case of a 66-year-old woman who developed acute onset left inner thigh intense burning pain radiating down the medial aspect of the left leg. Three days later, this was followed by a vesicular rash over the same distribution. By day 10, she started dragging her left leg and was unable to cross the left leg over the right leg. The weakness reached a nadir within three days and was accompanied by visible thigh atrophy. The intense pain was not relieved with hydrocodone 10 milligrams (mg) twice daily, pregabalin 150 mg twice daily and a trial of a lumbar epidural. The rash gradually resolved with a course of famciclovir 800 mg three times daily for seven days. The weakness persisted for three months, after which she was referred to neurology. By then, she had lost 10 pounds in weight and was struggling to stand up from the seated position and walk with confidence. Her past medical history was remarkable for hypertension treated with lisinopril 10 mg daily. She was a non-smoker and did not drink alcohol.

On examination, blood pressure (BP) was 123/75, with a pulse of 100 beats per minute. Her weight was 205 pounds, height of 5-foot and 9-inches with a body-mass-index (BMI) of 30.3.

The neurological examination revealed a gait characterized by a lag of the left leg when she made a stride. Heel and toe-standing was preserved. She was unable to stand up from the seated position with the arms folded.

Motor examination revealed profound weakness of the left ilio-psoas with moderate weakness of the left quadriceps and left hip adduction and obvious left thigh atrophy. The power in the lower extremities was graded with the Medical Research Council (MRC) grading scale (Table 1).

**Table 1:** MRC grading of muscle groups in both legs. MRC=Medical Research Council

	RIGHT LEG	LEFT LEG
Ilio-psoas	5	2
Hip adduction	5	3
Hip abduction	5	5
Quadriceps	5	3
Hamstrings	5	5
Ankle dorsi-flexion	5	5
Ankle plantar-flexion	5	5

**Figure 1:**
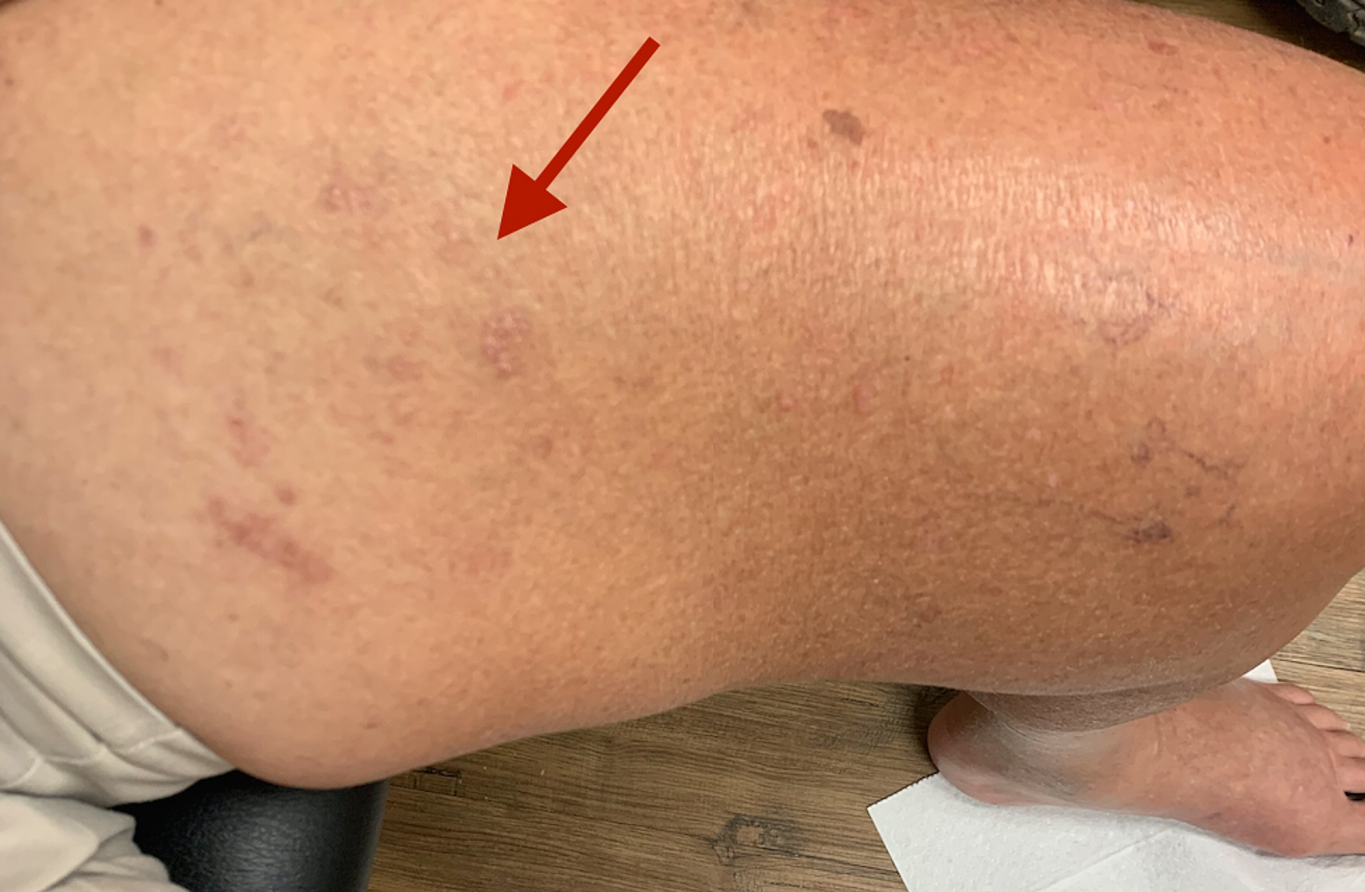
Left inner thigh: resolving shingles rash (red arrow).

Sensory examination revealed exquisite tenderness to touch over the left medial thigh. Deep tendon reflexes displayed a lively right knee jerk, an absent left knee and bilateral absent ankle jerks. The healed rash of shingles is demonstrated over the left inner thigh, dermatomes L2 and L3 (Figure 1). 

**Figure 2:**
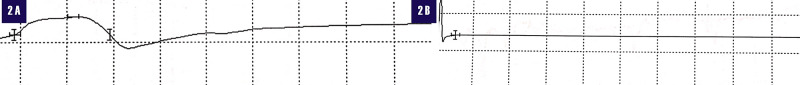
Nerve conduction study of femoral motor nerve with recording from the rectus femoris muscle. 2A: normal right femoral motor amplitude. 2B: absent left femoral motor amplitude NCS: Nerve Conduction Study

A nerve conduction study revealed an absent left femoral motor amplitude when recording from the left rectus femoris muscle (Figure 2).

The left sural and left peroneal sensory amplitudes were reduced with normal velocities. The left peroneal and left tibial motor amplitudes and velocities were normal for age.

**Figure 3:**
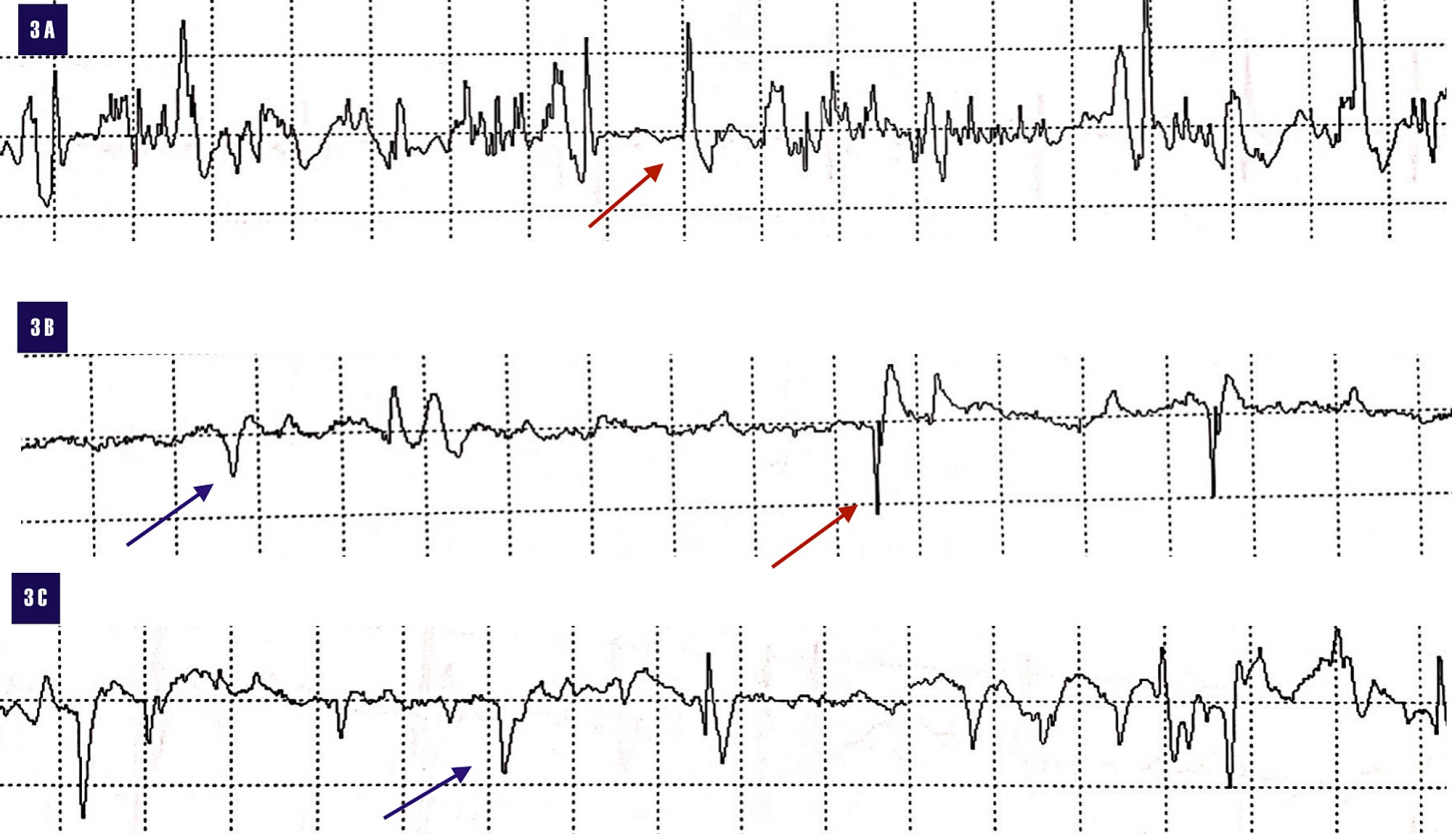
Electromyogram demonstrating acute florid denervation of 3A. Left ilio-psoas. 3B. Left vastus lateralis and 3C. Left adductor longus. Fibrillation potentials (red arrows). Positive sharp waves (blue arrows). EMG: Electromyography

The electromyogram revealed severe acute denervation of the left ilio-psoas, left vastus lateralis and left adductor longus with reduced recruitment of all three muscle groups (Figure 3).

There was no evidence of lumbar paraspinal denervation. This spectrum of findings of an absent femoral nerve amplitude and acute denervation of the left ilio-psoas, left vastus lateralis and left adductor longus muscles is quite typical of a lumbar plexopathy.

Due to the profound nature of the left proximal leg weakness, a magnetic resonance imaging study of the lumbar spine and lumbar plexus was obtained. This did not reveal any significant neuroforaminal stenosis or lumbar plexus enhancement, noting that the imaging studies were obtained at least three months after the onset of her symptoms. 

Due to her disability from the weakness, the patient was treated with intravenous methylprednisolone 500 mg weekly for four weeks. Duloxetine 60 mg daily was also added for pain control. Five weeks later, the patient was re-evaluated. The cadence of her gait and stride had improved with increased confidence of stance. The power of her left proximal leg had improved dramatically (Table 2).

**Table 2:** Dramatic improvement of left proximal leg weakness using MRC scale. MRC= Medical Research Council Scale

	Pre-steroid infusion	Post-steroid infusion
Left hip flexion	2	4
Left knee extension	3	5
Left hip adduction	3	5

The burning dysesthesia of the left inner thigh had also subsided significantly. 

## Discussion

The parallels between post-HSP and diabetic radiculo-plexopathy are striking. What is lacking for post-HSP are pathological studies such as sural nerve biopsies and cerebrospinal fluid studies. However, as outlined earlier, these diseases are similar phenotypically.

The microvaculitis of diabetic radiculo-plexopathy is well documented with transmural small blood vessel inflammation, epineural peri-blood vessel inflammation, hemo-siderin laden macophages in the blood vessel wall and multi-focal nerve fiber loss implying an ischemic etiology [13]. 

It is well established that VZV causes an arteriopathy that can lead to ischemic strokes, hemorrhagic strokes and frank vasculitis. It is postulated that the VZV travels retrogradely from the dorsal root ganglia and trans-axonally into the adventitia of the vasa nervorum and invades the tunica media and intima, where it triggers an intense inflammatory response. This leads to duplication of the internal elastic lamina and loss of smooth muscle cells of the tunica media. These processes can lead to intra-luminal thrombosis and friability of the blood vessels, which can lead to a hemorrhagic tendency [16,17,18]. 

The invasiveness of arterial blood vessels by VZV was spectacularly demonstrated by Gilden et al. in patients with temporal arteritis. VZV antigen was found in the adventitia, tunica media and tunica intima in descending order of frequency in an overwhelming number of patients with giant cell arteritis-positive superficial temporal arteries [19]. 

In an update on VZV vasculopathy, Nagel et al. emphasizes the pervasive and pernicious nature of VZV, sparing very few arterial territories, and involving the spinal cord vasculature, peripheral arterial vessels, posterior ciliary arteries and ophthalmic arteries, potentially explaining the anterior ischemic optic neuropathy of giant cell arteritis [20]. 

When clinically indicated, especially with disabling weakness and without contraindications, the rationale for a therapeutic trial of an immunomodulatory agent is justified in patients with post-HSP. The inflammatory phenotype (acuity, subacute progression, pain, weight loss) and its parallel semiology with established vasculitic diseases are too conspicuous to ignore. Future studies may be directed at more imaging studies (plexuses), cerebrospinal fluid analysis, serum biomarkers of inflammation and sural nerve biopsies. Randomized controlled trials are ideal, but the rarity of this condition may preclude such a possibility.

## Conclusions

Post-HSP is a fascinating disorder. It's similar features to diabetic radiculo-plexopathy, specifically its acuity, subacute progression and painful nature are noteworthy and underlie its inflammatory nature . However, we know that post-HSP has an infectious etiology, namely the VZV. Furthermore, the dramatic rash antedating the weakness exposes the neurologic localization of the inciting lesion. In our case, an L2 and L3 dermatomal rash concurs with weakness involving the femoral- and obturator nerve-innervated muscles, both confirmed clinically and electrophysiologically. The pathological data of VZV-induced neurological disease is dramatic, revealing a frank vasculitis as in giant-cell temporal arteritis and cerebral VZV-induced arteritis, to quote two examples. It therefore stands to reason to treat patients with a combination of anti-virals and immunomodulatory therapy, when indicated. Needless to say, randomized controlled clinical trials would be ideal, but given the rarity of this condition, we may have to settle with case reports. Therefore, the importance of our observation of dramatic improvement of a case of severe post-HSP with high-dose intravenous pulse solumedrol needs to be disseminated. 
